# Maternal Microbial Reservoirs Are Associated with Early Bacterial and Archaeal Community Assembly in Neonatal Hu Lambs

**DOI:** 10.3390/ani16121862

**Published:** 2026-06-17

**Authors:** Bingbing Huang, Chunxia Mao, Taojie Xu, Shaoshi Ji, Li He, Ping Sheng

**Affiliations:** Jiangxi Functional Feed Additive Engineering Laboratory, Institute of Biomanufacturing, Jiangxi Academy of Sciences, Nanchang 330095, China; bbhuang217@163.com (B.H.);

**Keywords:** Hu sheep, colostrum, vaginal microbiota, amniotic fluid, gut microbiota, 16S rRNA sequencing, archaea

## Abstract

Early-life microbial community assembly is important for gastrointestinal development, immune maturation, and feed utilization in young ruminants, but the maternal reservoirs associated with neonatal lamb gut microbial profiles remain insufficiently defined. This exploratory study characterized bacterial and archaeal communities in maternal vaginal secretions, amniotic fluid, and colostrum, together with rectal feces from Hu lambs at birth and at 5 days of age, using 16S rRNA gene amplicon sequencing in six matched ewe–lamb pairs. Alpha-diversity differences were evaluated using the Kruskal–Wallis test followed by pairwise Wilcoxon rank-sum tests where appropriate, beta-diversity was assessed by principal coordinate analysis based on Bray–Curtis distance, and discriminatory taxa were identified using LEfSe; statistical significance was defined as *p* < 0.05. Microbial community structure differed among sample types. Feces collected at birth showed low bacterial richness and a distinct community profile, whereas feces from 5-day-old lambs displayed higher bacterial richness and a more complex taxonomic composition. Shared-ASV and LEfSe analyses suggested that vaginal-associated taxa were more closely associated with the initial fecal microbiota at birth, while colostrum-associated taxa were more evident in day-5 feces. Archaeal communities showed weaker separation than bacterial communities, but methanogenic lineages became more distinguishable by day 5. These findings suggest that early gut microbial assembly in Hu lambs is associated with multiple maternal reservoirs and rapid postnatal ecological selection. However, due to the limited sample size, short observation period, lack of formal source-tracking analysis, and absence of low-biomass negative controls, the results should be interpreted cautiously as preliminary associations rather than definitive evidence of vertical transmission.

## Simple Summary

Newborn lambs are exposed to microbes from several maternal and environmental sources. Understanding the early assembly of these communities is important because the neonatal gut microbiota may influence digestion, immune development, and later growth. This exploratory study examined microbial communities in the vagina, amniotic fluid, and colostrum of Hu sheep ewes, together with rectal feces from their lambs at birth and five days later. The gut microbial community of newborn lambs differed strongly from that observed after five days, suggesting rapid postnatal restructuring. Microbial signatures related to the maternal reproductive tract were more evident in feces collected at birth, whereas colostrum-associated bacteria were more closely associated with the fecal community at day 5. Archaeal microbes, including methanogenic lineages, appeared to establish more slowly. Because of the small sample size and the lack of low-biomass negative controls, these findings should be interpreted as preliminary associations rather than direct evidence of maternal microbial association.

## 1. Introduction

Early life is a critical window for microbial community assembly, during which the neonatal gastrointestinal tract is rapidly exposed to maternal and environmental microorganisms that may influence host physiology beyond the immediate postnatal period [1,2]. In ruminants, this process is especially important because microbial communities contribute to intestinal maturation, immune development, nutrient utilization, and later digestive efficiency [1,3]. Recent studies in lambs and other young ruminants have further associated early gut microbial assembly with subsequent growth performance, highlighting this developmental window as a relevant target for animal production and sustainable livestock management [1]. Hu sheep are an important indigenous Chinese sheep breed known for high fecundity, strong adaptability, good maternal performance, and suitability for intensive production systems. Therefore, understanding early gut microbial assembly in Hu lambs may provide useful information for improving neonatal health and management in sheep production.

The gastrointestinal microbiota is a major regulator of host homeostasis. It supports epithelial barrier function, immune maturation, pathogen resistance, and the conversion of dietary substrates into bioavailable metabolites [1,3]. For ruminants, these microbial functions are particularly important because the host depends extensively on microbial metabolism to obtain usable energy and nutrients from feed. Studies in neonatal lambs, goat kids, and calves indicate that communities established during the first days of life can influence later microbial succession, intestinal function, and host performance [1,4].

Maternal microbial exposure is increasingly recognized as a potential contributor to neonatal gut microbiota establishment [2,3]. Before, during, and shortly after birth, offspring may encounter maternal microorganisms through the reproductive tract, amniotic fluid, the birth canal, colostrum, and milk [2,3,5]. Among these potential reservoirs, vaginal microbiota may provide direct exposure during delivery, whereas colostrum supplies both nutrients and microorganisms after birth [2,3]. Recent 16S rRNA gene sequencing evidence in ewes has shown that the vaginal microbiota changes throughout gestation and contains bacterial taxa that may be relevant to periparturient microbial exposure, further supporting the reproductive tract as a plausible maternal microbial source for newborn lambs [6]. In calves, source-tracking studies have linked neonatal fecal microbiota to maternal vaginal secretions and colostrum during the first week of life, although later assembly is increasingly affected by environmental and host-associated selection [2].

The contribution of amniotic fluid to prenatal or perinatal microbial exposure remains debated [4]. Although microbial signals in fetal and perinatal compartments are biologically plausible, amniotic fluid is a low-biomass sample type and is therefore particularly vulnerable to contamination during sampling, DNA extraction, and sequencing [4,7,8]. Recent consensus recommendations for low-biomass microbiome studies emphasize the need for rigorous contamination prevention, negative controls, and transparent reporting, while critical evaluations of the fetal microbiome field have highlighted the risk of overinterpreting weak microbial signals in prenatal samples [7,8]. Consequently, microbial signals detected in amniotic fluid should be interpreted cautiously and ideally evaluated together with other maternal and neonatal compartments.

Most ruminant microbiome studies have focused on rumen development, dietary modulation, or microbial shifts later in life. The immediate perinatal period remains less well characterized, particularly in matched dam–offspring studies that simultaneously assess multiple maternal reservoirs and neonatal intestinal samples. This gap limits our ability to reconstruct plausible microbial association routes during the earliest stages of lamb gut microbial assembly [5].

Neonatal gut communities undergo rapid restructuring after birth [5]. In goat kids, fecal microbiota follows clear age-dependent succession, with high early Proteobacteria abundance and later stabilization of *Firmicutes*- and *Ruminococcaceae*-associated communities [5]. Similarly, meconium and early fecal samples from calves are often enriched in facultative anaerobes, whereas subsequent development reflects dietary exposure, host selection, and ongoing colonization from maternal and environmental sources [2,5]. These findings indicate that the earliest neonatal microbiota is transitional rather than stable.

Despite growing interest in maternal microbial contribution, two knowledge gaps remain relevant to newborn lambs. First, the associations of the maternal vagina, amniotic fluid, and colostrum with early lamb gut microbial assembly have not been systematically compared in a matched ewe–lamb design [2,5]. Second, bacterial and archaeal communities may follow distinct developmental trajectories after birth, yet these domains are rarely evaluated in parallel during the neonatal period [9]. This distinction is important because methanogenic archaea may differ from bacteria in ecological dependence, timing of establishment, and relevance to later digestive function and methane biology [9].

Clarifying early microbial sources and succession can improve understanding of host–microbe co-development during a foundational stage of life [3]. From an applied perspective, identifying maternal reservoirs that are associated with neonatal microbial profiles may inform microbiome-oriented strategies, including maternal management, hygienic perinatal handling, and optimized colostrum use to support neonatal health and performance [5]. Such interventions may be especially influential during early life because the neonatal microbiome is more plastic than the adult microbiome [3]. In Hu sheep, maternal rumen bacteriota has been shown to influence offspring rumen bacteriota and developmental traits, suggesting that mother–offspring microbial associations may be relevant to young ruminant development beyond single gut compartments [10]. However, whether multiple maternal reservoirs, including the vagina, amniotic fluid, and colostrum, are associated with early intestinal microbial assembly in neonatal Hu lambs remains unclear.

**Figure 1:**
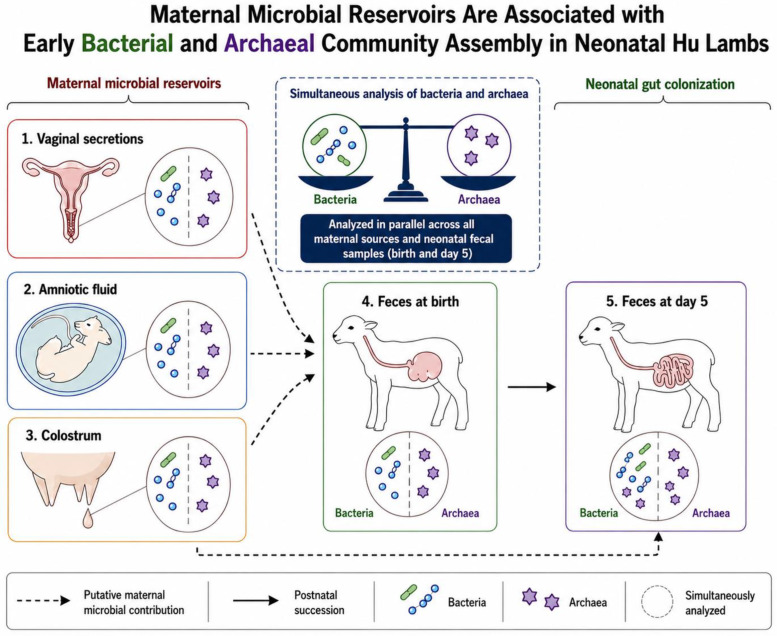
Schematic summary model of study.

In this exploratory study, we characterized microbial communities in maternal vaginal secretions, amniotic fluid, and colostrum, together with rectal feces from Hu lambs at birth and at 5 days of age (Figure 1). Using 16S rRNA gene amplicon sequencing, we compared microbial overlap, alpha diversity, beta diversity, taxonomic composition, and discriminatory taxa across maternal and neonatal sample types. We hypothesized that the early gut microbial profile of lambs is associated with multiple maternal reservoirs rather than a single source and that this community undergoes rapid restructuring during the first days after birth. We further hypothesized that bacterial and archaeal communities would exhibit distinct early assembly patterns, reflecting different ecological dynamics.

## 2. Materials and Methods

### 2.1. Animals and Experimental Design

The experiment was conducted at the Taihe County Animal Testing Base in Jiangxi Province, China. Ten clinically healthy Hu sheep ewes aged 2–4 years and with similar expected delivery dates were initially selected from the same flock. Ewes were included based on normal health status, comparable gestational stage, absence of obvious clinical disease during late pregnancy, and no documented antibiotic or probiotic treatment during the periparturient sampling period. During the experimental period, all ewes were housed in the same barn under identical management conditions, with ad libitum access to feed and water. During the first five days, lambs remained with their dams under the same farm management conditions. They suckled naturally and were housed in the same barn environment. The composition of the basal diet is shown in Table 1, and the diet was formulated to meet the nutritional requirements of late-pregnant ewes. The barn was naturally ventilated, cleaned regularly, and managed consistently throughout the experimental period. Approximately 1 week before the expected lambing date, the ewes were transferred to the delivery room and monitored by trained personnel. A matched ewe–lamb pair was defined as one lamb and its biological dam, confirmed by direct observation of delivery and individual animal identification records. Pairs were retained when the ewe and lamb were clinically healthy, natural delivery occurred without obvious dystocia, rectal feces could be collected before colostrum intake, and complete maternal and neonatal sample sets were available. According to these predefined sampling criteria and sample availability, six matched ewe–lamb pairs were retained for microbiota analysis. The sample size was determined based on the number of eligible pregnant Hu ewes available at the experimental Research Base during the experimental period, and animal welfare considerations.

### 2.2. Sample Collection

All samples were collected immediately after delivery using sterile procedures. Sterile gloves, sterile swabs, sterile syringes, and sterile microcentrifuge tubes were used throughout sampling. Vaginal secretions were collected from the lateral vaginal wall and posterior fornix using sterile swabs while avoiding contact with feces, wool, skin, or the external perineal surface. For amniotic fluid sampling, when the amniotic sac was clearly visible, the fetal membrane surface was disinfected with 70% ethanol, and at least 10 mL of amniotic fluid was collected by puncture using a sterile 20-mL syringe. Rectal fecal samples at birth were collected before colostrum intake and before prolonged environmental exposure. Colostrum was collected immediately after delivery and before the first suckling under aseptic conditions. Before collection, the udder and teats were cleaned with soapy water, rinsed thoroughly with sterile saline, and dried with sterile gauze. The sampler wore sterile gloves, and sterile collection tubes were used throughout the procedure. The first few drops of colostrum were discarded to flush the teat canal and reduce potential contamination from the teat surface. Approximately 5 mL of colostrum was then collected directly into sterile tubes, while avoiding contact between the tube opening and the teat, skin, wool, or surrounding environment. All colostrum samples were immediately snap-frozen in liquid nitrogen and stored at −80 °C until DNA extraction.

### 2.3. DNA Extraction

All procedures were performed in physically separated sterile work areas to reduce cross-contamination, particularly because amniotic fluid and colostrum may represent relatively low-biomass sample types that are susceptible to contamination during microbiome analysis [7]. Genomic DNA was extracted from all samples using the cetyltrimethylammonium bromide (CTAB) method combined with bead-beating, a mechanical disruption step commonly used to enhance microbial cell lysis and reduce extraction bias among microbial taxa with different cell-wall structures [11,12]. DNA integrity and concentration were assessed by agarose gel electrophoresis, and DNA samples were diluted with sterile water to a final concentration of 1 ng/μL for downstream amplification. Extraction blanks, PCR negative controls, sequencing controls, and quantitative biomass measurements were not included in the present study; this limitation is explicitly considered in the Discussion.

### 2.4. PCR Amplification

Diluted genomic DNA was used as the template for PCR amplification of the 16S rRNA gene. For bacterial community profiling, the V3–V4 region of the bacterial 16S rRNA gene was amplified using barcode-tagged primers 338F (5′-ACTCCTACGGGAGGCAGCA-3′) and 806R (5′-GGACTACHVGGGTWTCTAAT-3′). For archaeal community profiling, archaeal 16S rRNA gene fragments were amplified using barcode-tagged primers Arch349F (5′-GYGCASCAGKCGMGAAW-3′) and Arch806R (5′-GGACTACVSGGGTATCTAAT-3′). PCR amplification was performed using a high-fidelity DNA polymerase to reduce amplification errors. PCR products were first verified by agarose gel electrophoresis to confirm successful amplification and the expected amplicon size. Qualified amplicons were purified to remove residual primers, primer dimers, nucleotides, enzymes, and other PCR components. Purified PCR products were then quantified, normalized according to their concentrations, and pooled in equimolar amounts to construct 16S rRNA amplicon sequencing libraries following standard amplicon library preparation procedures [13,14,15,16].

After sequencing and taxonomic assignment, bacterial and archaeal datasets were filtered separately. For the bacterial dataset, sequence features assigned to non-bacterial domains were removed, and Bacteria-assigned ASVs were retained for downstream analyses. For the archaeal dataset, sequence features assigned to non-archaeal domains were removed, and Archaea-assigned ASVs were retained for downstream analyses. Alpha diversity, beta diversity, taxonomic composition, and LEfSe analysis were then performed separately for the bacterial and archaeal datasets.

### 2.5. High-Throughput Sequencing

Constructed libraries were subjected to quality control, and qualified libraries were sequenced on the Illumina NovaSeq 6000 platform (Illumina, San Diego, CA, USA).

### 2.6. Bioinformatics and Statistical Analysis

Raw reads from the bacterial and archaeal amplicon datasets were processed independently using the same bioinformatics workflow. Raw reads were first quality-filtered using Trimmomatic v0.33 [17]. Primer sequences were identified and removed using Cutadapt v1.9.1 [18] to generate clean reads. Sequence quality control, paired-end read merging, denoising, and chimera removal were performed using the DADA2 algorithm implemented in QIIME2 v2020.6 [19,20]. DADA2 was used to infer amplicon sequence variants (ASVs), and all downstream analyses were conducted at the ASV level. Representative ASV sequences were taxonomically assigned using a Bayesian classifier against the SILVA 138 SSU rRNA database [21,22].

After taxonomic assignment, non-target-domain ASVs were filtered out from each dataset. For the bacterial amplicon dataset, ASVs assigned to non-bacterial domains were removed, and Bacteria-assigned ASVs were retained for bacterial community analysis. For the archaeal amplicon dataset, ASVs assigned to non-archaeal domains were removed, and Archaea-assigned ASVs were retained for archaeal community analysis. The bacterial and archaeal ASV datasets were then analyzed separately for diversity, taxonomic composition, Venn analysis, and LEfSe analysis.

Alpha- and beta-diversity analyses were performed after rarefaction to 50,021 reads per sample for the bacterial dataset and 40,129 reads per sample for the archaeal dataset, corresponding to the minimum number of non-chimeric reads retained among the samples included in each dataset. Rarefaction curves were generated to evaluate sequencing depth and community richness. Venn diagrams were produced using the VennDiagram package in R v3.0.3. The Shannon and Chao1 indices were calculated to evaluate alpha diversity. Differences among groups were assessed using the Kruskal–Wallis test followed by pairwise Wilcoxon rank-sum tests where appropriate; *p* < 0.05 was considered statistically significant. Exact *p* values are shown where available. Principal coordinate analysis (PCoA) based on Bray–Curtis distance was used to evaluate community structural differences among sample types [23]. LEfSe analysis was performed to identify putative discriminatory taxa among groups. The Kruskal–Wallis test was used to detect taxa with significant inter-group differences, and Wilcoxon rank-sum testing was applied where appropriate to evaluate the consistency of differences. Taxa with *p* < 0.05 and an LDA score >4.0 were retained as putative discriminatory taxa. Considering the limited sample size, LEfSe-derived taxa were interpreted as exploratory microbial signatures rather than definitive biomarkers. No formal source-tracking analysis, such as SourceTracker or FEAST, was performed; therefore, microbial overlap was interpreted as association rather than direct transmission.

## 3. Results

The results are presented below according to microbial overlap, alpha diversity, taxonomic composition, beta diversity, and discriminatory taxa.

### 3.1. Shared and Unique ASVs

**Figure 2:**
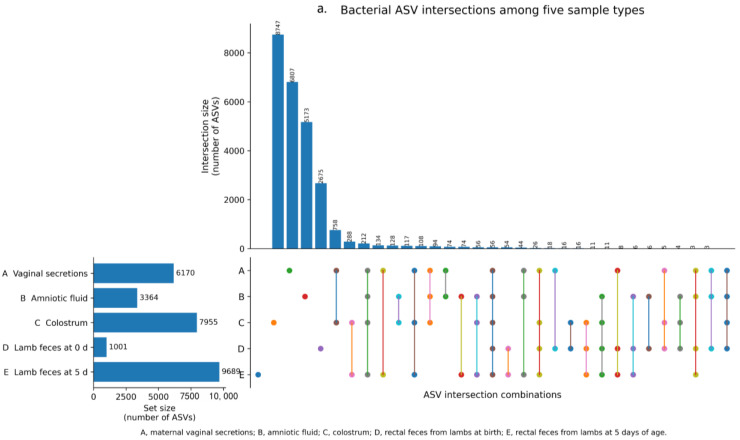

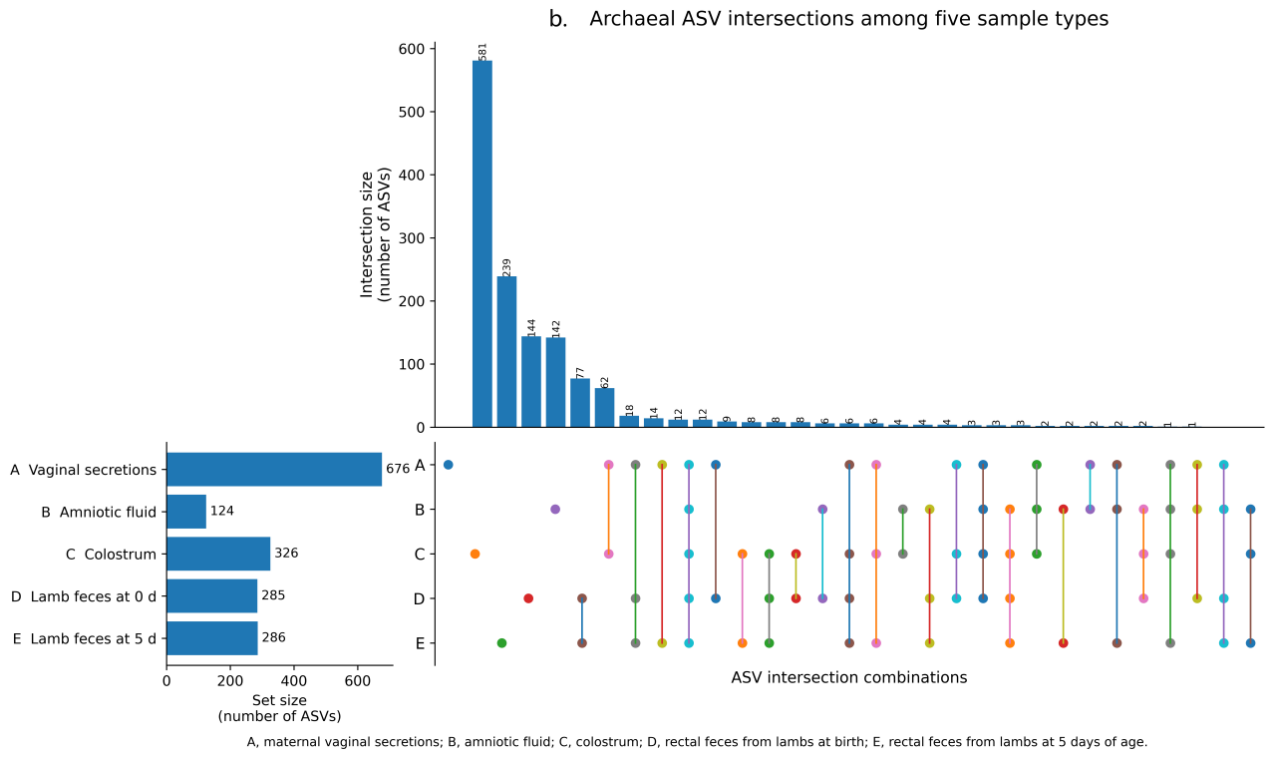
UpSet plots showing shared and group-specific bacterial and archaeal ASVs among maternal and neonatal sample types.

UpSet analysis revealed both shared and sample-specific microbial ASVs among the five sample types, including maternal vaginal secretions, amniotic fluid, colostrum, and rectal feces from lambs at birth and at 5 days of age (Figure 2). Shared and group-specific ASVs were calculated based on a presence/absence ASV matrix, and ASVs detected in at least one sample within each group were included in the group-level intersection analysis. For bacteria, feces from 5-day-old lambs contained the largest number of group-specific ASVs (8747), followed by colostrum (6807), vaginal samples (5173), amniotic fluid (2675), and feces collected at birth (758). A total of 56 bacterial ASVs were shared across all five sample types, indicating limited but detectable overlap among maternal and neonatal compartments. For archaea, vaginal samples harbored the highest number of group-specific ASVs (581), followed by colostrum (239), feces from 5-day-old lambs (144), feces collected at birth (142), and amniotic fluid (77). Only 12 archaeal ASVs were shared across all five groups. These descriptive UpSet results indicate pronounced sample-type-specific microbial profiles across maternal and neonatal samples. Inferential statistical comparisons of microbial richness and community structure among groups were further evaluated using alpha-diversity tests and beta-diversity analysis, respectively, as described below.

### 3.2. Alpha Diversity Across Maternal and Neonatal Samples

**Figure 3:**
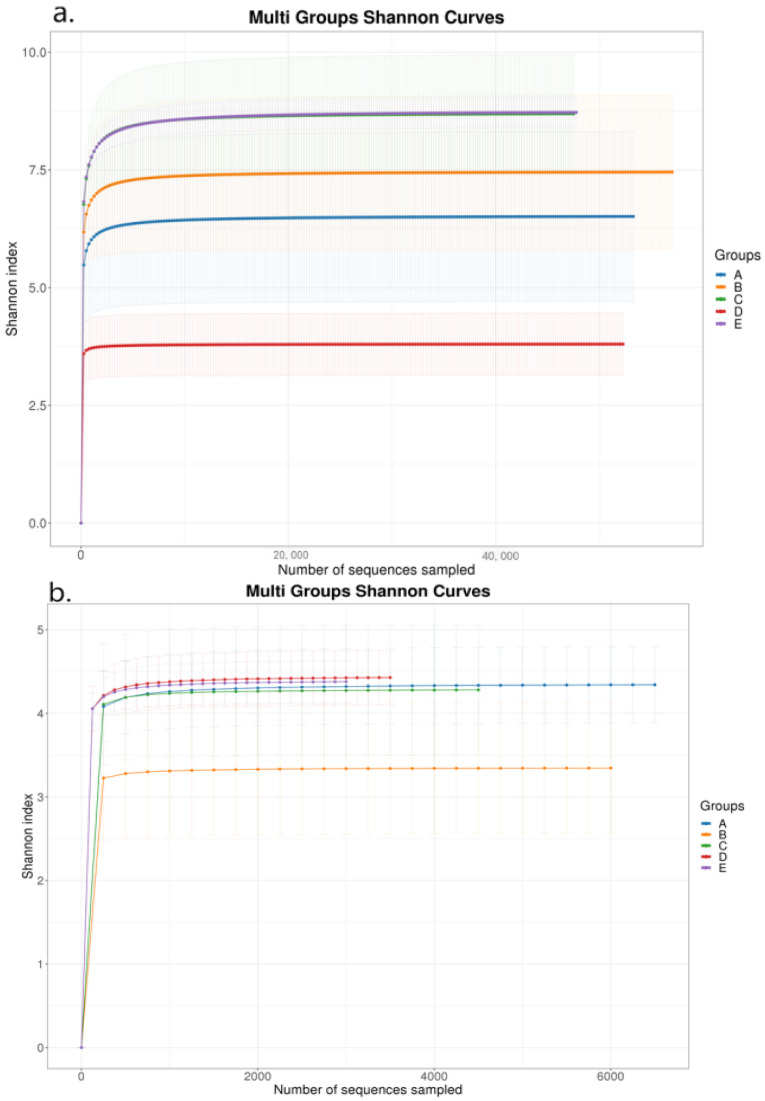
Shannon rarefaction curves for bacterial (**a**) and archaeal (**b**) communities. A, vaginal secretions; B, amniotic fluid; C, colostrum; D, feces from lambs at birth; E, feces from lambs at 5 days of age.

**Figure 4:**
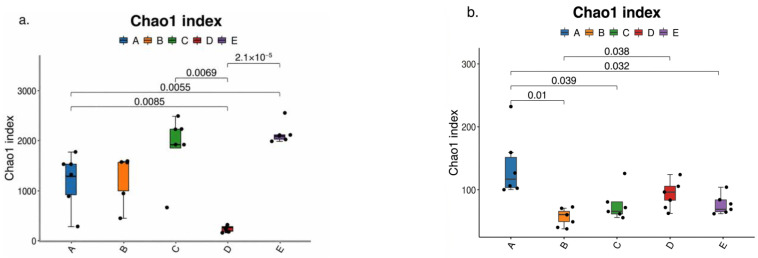
Chao1 richness estimates for bacterial (**a**) and archaeal (**b**) communities. A, vaginal secretions; B, amniotic fluid; C, colostrum; D, feces from lambs at birth; E, feces from lambs at 5 days of age. The center line represents the median, the box represents the interquartile range, whiskers represent 1.5× interquartile range, and individual points represent biological samples. Statistical significance was determined using the Kruskal–Wallis test, *p* values are shown above the corresponding comparisons.

Alpha-diversity analysis revealed distinct developmental patterns for bacterial and archaeal communities in neonatal lambs. Rarefaction curves approached saturation in all groups, indicating sufficient sequencing depth for the observed communities (Figure 3). Bacterial diversity and richness increased markedly after birth, with feces from 5-day-old lambs showing the greatest community complexity, whereas feces collected at birth showed the lowest bacterial richness. In contrast, archaeal diversity remained comparatively limited and changed only modestly among sample types. Chao1 estimates further supported these patterns, showing significant postnatal expansion of bacterial richness but a more restricted archaeal assembly process (Figure 4). Together, these findings suggest that the neonatal gut undergoes rapid bacterial community expansion during the first 5 days of life, whereas archaeal community assembly remains less mature.

### 3.3. Microbial Composition of Maternal and Neonatal Samples

**Figure 5:**
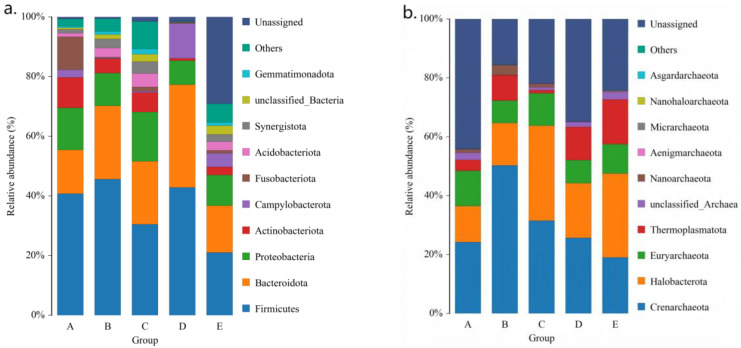
Relative abundances of bacterial (**a**) and archaeal (**b**) taxa at the phylum level. A, vaginal secretions; B, amniotic fluid; C, colostrum; D, feces from lambs at birth; E, feces from lambs at 5 days of age.

Phylum-level taxonomic profiling revealed marked differences in microbial composition among maternal and neonatal sample types (Figure 5). In bacterial communities, *Firmicutes* was the predominant phylum across all groups, followed by *Bacteroidota* and *Proteobacteria*, although their relative abundances varied by sample type. Feces collected at birth showed a distinct profile, including a relatively greater contribution of *Campylobacterota*, whereas feces from 5-day-old lambs exhibited a more diverse phylum-level structure. In archaeal communities, the major phyla included *Crenarchaeota*, *Halobacterota*, and *Euryarchaeota*, whereas *Thermoplasmatota* was relatively more abundant in neonatal fecal samples. These results demonstrate clear compartment- and age-associated differences in microbial composition during early-life gut microbial assembly.

**Figure 6:**
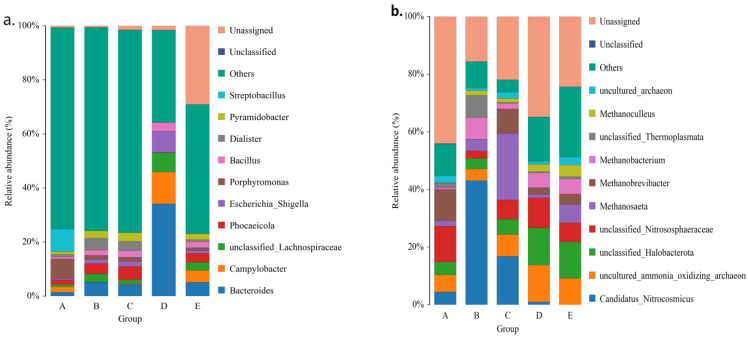
Relative abundances of bacterial (**a**) and archaeal (**b**) taxa at the genus level. A, vaginal secretions; B, amniotic fluid; C, colostrum; D, feces from lambs at birth; E, feces from lambs at 5 days of age.

Genus-level taxonomic profiling also revealed marked differences in microbial composition among maternal and neonatal sample types (Figure 6). In bacterial communities, *Bacteroides* predominated in amniotic fluid, colostrum, and feces from 5-day-old lambs, whereas vaginal samples were enriched in *Porphyromonas* and *Streptobacillus*. By contrast, feces collected at birth showed a distinct bacterial profile, supporting rapid restructuring of the gut microbiota during the early postnatal period. In archaeal communities, the dominant genera included *Methanobrevibacter*, *Methanosaeta*, and *Methanobacterium*, and methanogenic archaea were more abundant in feces from 5-day-old lambs than in feces collected at birth. These results demonstrate pronounced genus-level niche specificity and progressive microbial maturation during early-life gut microbial assembly.

### 3.4. Beta Diversity Across Maternal and Neonatal Sample Types

**Figure 7:**
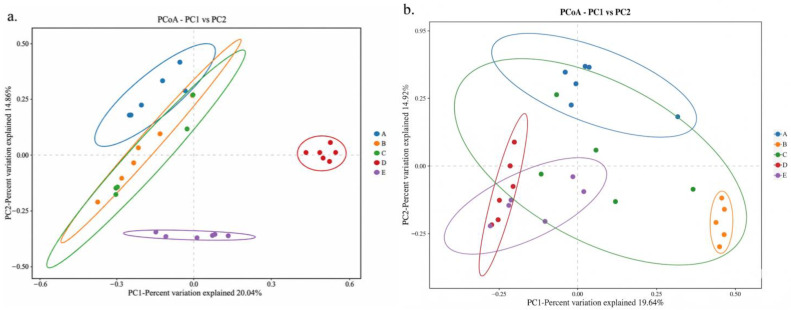
Principal coordinate analysis (PCoA) based on Bray–Curtis distance for bacterial (**a**) and archaeal (**b**) communities. A, vaginal secretions; B, amniotic fluid; C, colostrum; D, feces from lambs at birth; E, feces from lambs at 5 days of age.

PCoA based on Bray–Curtis distance revealed differences in microbial community structure among maternal and neonatal sample types (Figure 7). For bacterial communities, the first two principal coordinates explained 34.90% of the total variation, with PC1 and PC2 explaining 20.04% and 14.86%, respectively. Feces collected at birth formed a relatively distinct cluster, and feces from 5-day-old lambs also showed separation from birth fecal samples, suggesting rapid postnatal restructuring of the bacterial community. Vaginal samples showed partial clustering, whereas amniotic fluid and colostrum exhibited some overlap with other maternal and neonatal groups.

In contrast, archaeal communities showed weaker separation and greater overlap among sample types than bacterial communities, indicating a less pronounced structural shift during the early neonatal period. Although neonatal fecal samples showed some separation from maternal samples, the overall clustering pattern was less discrete for archaea than for bacteria. These results suggest that bacterial communities underwent more pronounced postnatal structural differentiation than archaeal communities during the first 5 days of life. Because the first two PCoA axes captured only part of the total variation, these ordination plots should be interpreted as two-dimensional visualizations of community differences rather than complete representations of all beta-diversity variation.

### 3.5. Discriminatory Taxa Identified by LEfSe Analysis

**Figure 8:**
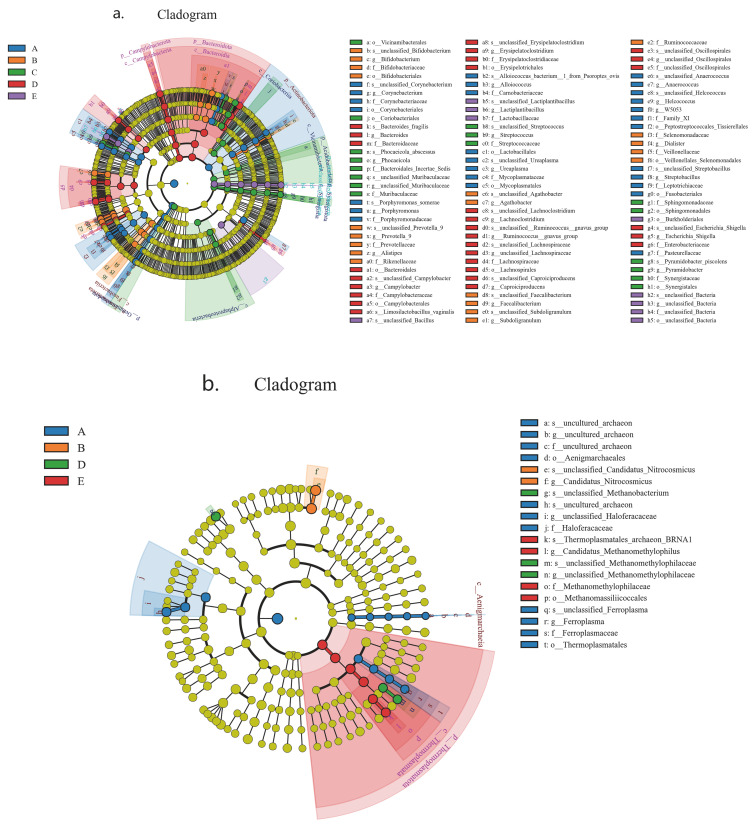
LEfSe cladograms for bacterial (**a**) and archaeal (**b**) discriminatory taxa. A, vaginal secretions; B, amniotic fluid; C, colostrum; D, feces from lambs at birth; E, feces from lambs at 5 days of age. LDA, linear discriminant analysis; LEfSe, linear discriminant analysis effect size.

LEfSe analysis (LDA > 4.0) identified distinct bacterial signatures for each group (Figure 8). Vaginal samples were enriched in *Corynebacterium* and *Corynebacteriaceae*. Feces from lambs at birth showed discriminatory taxa including *Campylobacterota*, *Erysipelatoclostridiaceae*, and *Erysipelatoclostridium*, which may reflect early microbial exposure associated with delivery. Colostrum was enriched in *Vicinamibacterales*, *Bifidobacterium*, and *Streptococcus*. Feces from 5-day-old lambs harbored discriminatory taxa including *Ruminococcaceae* and *Oscillospira*, suggesting that the day-5 fecal community was more closely associated with postnatal microbial succession and colostrum-related inputs than with the bacterial profile observed at birth. Amniotic fluid showed relatively few discriminatory taxa, indicating a comparatively weaker detectable signal in relation to lamb gut microbial assembly in the present dataset.

Archaeal LEfSe analysis identified discriminatory taxa in the vagina, amniotic fluid, feces at birth, and feces at 5 days of age, whereas no colostrum-specific archaeal biomarker was detected. Vaginal samples were enriched in several uncultured archaeal lineages together with *Aenigmarchaeales*- and *Halobacteriaceae*-related taxa. Amniotic fluid was characterized mainly by *Candidatus Nitroscosmicus*-related lineages. Feces collected at birth showed enrichment of *Methanobacterium*- and *Methanomethylophilaceae*-related taxa, whereas feces from 5-day-old lambs were enriched in methanogenic lineages including *Candidatus Methanomethylophilus*, *Methanomethylophilaceae*, and *Methanomassiliicoccales*. These findings suggest that archaeal signals are detectable at birth but become more distinct by day 5, supporting a delayed and progressive establishment pattern for methanogenic archaea during early postnatal development.

## 4. Discussion

This exploratory study evaluated whether the early gut microbial profile of Hu lambs is associated with multiple maternal microbial reservoirs and whether bacterial and archaeal communities follow distinct postnatal assembly trajectories. Detectable microbial overlap was observed among the maternal vagina, amniotic fluid, colostrum, and neonatal feces, and the neonatal gut microbiota underwent marked restructuring during the first 5 days after birth. Together, these findings suggest that early gut community assembly in lambs involves exposure to multiple maternal microbial sources, followed by rapid postnatal ecological selection and community remodeling [2,3,4,5]. However, because no formal source-tracking or strain-resolved analysis was performed, these results should be interpreted as associations rather than direct evidence of maternal microbial association.

A key finding was the divergence between bacterial and archaeal assembly during the earliest postnatal period. Bacterial richness and complexity increased markedly by day 5, whereas archaeal diversity remained comparatively limited and community differentiation was less pronounced. This pattern suggests that bacterial community expansion is a dominant early ecological process, whereas archaeal development may be driven by postnatal ecological selection, milk-derived substrates, and bacterial-mediated niche formation [9,24,25,26].

The marked shift in bacterial community structure between birth and day 5 indicates that the neonatal gut microbiota is not simply a passive reflection of maternal exposure. Feces collected at birth formed a distinct cluster and showed relatively low richness, whereas day-5 feces displayed greater bacterial complexity and a different taxonomic profile. At the phylum level, feces at birth showed a relatively greater contribution of *Campylobacterota*, whereas day-5 feces exhibited a more diverse bacterial composition. At the genus level, *Bacteroides* became more prominent in 5-day-old lambs. These findings are consistent with ecological succession from a transient birth-associated inoculum to a more nutritionally adapted early-life community [24,25,26,27].

The LEfSe results further support the stage- and source-associated nature of early microbial assembly. In bacterial communities, vaginal samples were enriched in taxa such as *Corynebacterium* and *Corynebacteriaceae*, whereas feces collected at birth were characterized by *Campylobacterota*, *Erysipelatoclostridiaceae*, and *Erysipelatoclostridium*. This pattern suggests that vaginal exposure during delivery may be associated with the initial microbial profile of newborn lambs [2,3,28,29]. In contrast, colostrum was enriched in *Bifidobacterium* and *Streptococcus*, whereas feces from 5-day-old lambs harbored *Ruminococcaceae* and *Oscillospira*, suggesting that the dominant maternal association may shift after birth from delivery-associated exposure toward colostrum-associated and postnatal gut-selected microbial inputs [2,3,27,29].

The archaeal LEfSe analysis refined this interpretation. Discriminatory archaeal taxa were identified in the vagina, amniotic fluid, feces at birth, and feces at day 5, whereas no colostrum-specific archaeal biomarker was detected. Vaginal samples were enriched in uncultured archaeal lineages together with *Aenigmarchaeales*- and *Halobacteriaceae*-related taxa, whereas amniotic fluid was characterized mainly by *Candidatus Nitroscosmicus*-related lineages. Feces collected at birth showed enrichment of *Methanobacterium*- and *Methanomethylophilaceae*-related taxa, while feces from 5-day-old lambs were enriched in *Candidatus Methanomethylophilus*, *Methanomethylophilaceae*, and *Methanomassiliicoccales*. These findings indicate that archaeal colonization is detectable at birth but becomes more distinct by day 5, supporting delayed and progressive establishment of methanogenic archaea [9,25]. The absence of a colostrum-specific archaeal biomarker should not be interpreted as evidence that colostrum lacks archaea; rather, it may reflect low biomass, weak group specificity, and the limits of amplicon-based detection [5,9].

This interpretation is consistent with recent syntheses of sheep early-life microbiota development and with calf studies showing that site-specific microbial communities can be detected at or shortly after birth [4,30,31]. In this context, the delayed maturation of archaeal communities observed here supports the view that microbial exposure and stable colonization are not equivalent processes during early-life microbial assembly [5,30,31].

The shared-ASV analysis further supports the view that maternal reproductive and lactational compartments provide biologically relevant microbial exposures to the neonatal lamb gut. The presence of shared bacterial and archaeal ASVs across maternal and neonatal sample types suggests microbial overlap, whereas the large number of sample-specific ASVs and the clear beta-diversity separation among sample types indicate strong niche specificity. Maternal contribution should therefore be interpreted not as wholesale transfer of intact microbial communities, but as selective exposure to partially overlapping microbial signatures that are subsequently filtered by the neonatal gut habitat [32,33].

Within this framework, the vagina, amniotic fluid, and colostrum represent plausible but non-equivalent reservoirs associated with early microbial assembly [32,33]. The relatively limited number of discriminatory taxa detected in amniotic fluid suggests that its detectable association with early gut assembly may be weaker or less stable than that of the vagina or colostrum. However, this interpretation should be made cautiously because amniotic fluid is a low-biomass sample type that is particularly sensitive to contamination and background DNA [5,7,8,34]. Therefore, the amniotic-fluid-associated signals observed here should be interpreted as suggestive microbial associations rather than definitive evidence of prenatal microbial colonization.

This study extends current knowledge by evaluating bacterial and archaeal communities within the same analytical framework. Most perinatal microbiome studies in ruminants have focused primarily on bacteria, despite the well-established importance of archaea in ruminant digestive ecology and later function [3,5,9,35]. The recent ruminant gut archaeome catalogue also shows that archaeal lineages, including members of *Methanobacteriaceae* and *Methanomethylophilaceae*, represent important components of the ruminant gastrointestinal microbiome [35], which is consistent with the methanogenic lineages detected in neonatal feces in the present study. The finding that archaeal communities remained comparatively immature within the first 5 days of life suggests that stable archaeal colonization may be constrained more by ecological dependency than by simple early exposure [9,24]. This distinction is important because microbial association and long-term colonization are related but not identical biological processes [3,9].

The taxonomic patterns identified here support a dynamic and stage-dependent model of maternal microbial association in neonatal lambs. Vaginal microbiota appeared to be more closely associated with the initial gut microbial profile at birth, whereas colostrum-associated bacterial taxa were more evident in the community structure observed at day 5. Together with previous evidence that maternal rumen bacteriota can shape offspring rumen bacteriota and developmental traits in Hu sheep [10], the present findings suggest that mother–offspring microbial associations in this breed may involve multiple maternal compartments and may begin during the earliest stages of postnatal life. These maternal reservoirs should therefore not be viewed as isolated transmission pathways, but as complementary and temporally changing inputs whose relative influence shifts as the neonatal gastrointestinal tract, feeding behavior, and inter-microbial interactions develop after birth [36,37].

The enrichment of *Bifidobacterium* and *Streptococcus* in colostrum aligns with the view that colostrum functions as both a nutritional source and a microbial inoculum for the neonatal gut [2,3]. Meanwhile, the overrepresentation of *Ruminococcaceae* and *Oscillospira* in feces from 5-day-old lambs reflects an early progression toward a more complex anaerobic gut ecosystem typical of ruminants [4,24]. Although 16S rRNA gene sequencing cannot confirm direct functional activity, these taxonomic shifts are consistent with the concept that postnatal feeding and maternal milk-associated microbes contribute to maturation of the neonatal gut microbiota [27,37]. This interpretation is relevant from a production perspective because early gut microbial assembly has been linked to subsequent growth performance in suckling lambs [1].

Several limitations should be considered. First, the study included only six matched ewe–lamb pairs, and the short observation window captured only the first 5 days of life. Therefore, the present study should be regarded as exploratory, and the limited sample size may affect the robustness of LEfSe results, beta-diversity interpretation, and microbial overlap analyses. Second, 16S rRNA gene amplicon sequencing cannot provide strain-level evidence of direct vertical transmission, functional persistence, or stable colonization. Third, archaeal profiles may be influenced by primer bias, database coverage, and the absence of qPCR-based absolute quantification. Therefore, the archaeal results should be interpreted as relative archaeal community profiles detected under the present amplification and classification conditions, rather than a complete characterization of the neonatal lamb archaeome. Fourth, no formal source-tracking analysis, such as SourceTracker or FEAST, was performed; therefore, microbial similarity and overlap should be interpreted as association rather than direct transmission. Fifth, low-biomass samples such as amniotic fluid and colostrum require cautious interpretation. Although strict sterile sampling and physically separated laboratory procedures were used, extraction blanks, PCR negative controls, sequencing controls, mock communities, decontamination analysis, and quantitative biomass assessment were not included. Consequently, contamination and background DNA cannot be fully excluded, particularly for amniotic-fluid-associated signals [7,8]. Future studies should include larger longitudinal cohorts, rigorous low-biomass controls, formal source-tracking, strain-resolved metagenomics, and functional analyses to distinguish microbial exposure from stable colonization. From an applied perspective, the present findings suggest that maternal reproductive health, colostrum quality, early feeding, and perinatal hygiene may all influence neonatal gut microbial assembly [3,32].

## 5. Conclusions

Early gut microbial assembly in Hu lambs appears to be a multi-source process with rapid remodeling. Maternal vaginal secretions, amniotic fluid, and colostrum showed overlapping but non-identical microbial signatures, while bacterial and archaeal communities followed distinct early developmental trajectories. Vaginal-associated taxa were more closely associated with the fecal microbiota at birth, whereas colostrum-associated taxa were more evident in the community observed at day 5. For archaea, methanogenic lineages became more clearly differentiated by day 5, supporting a delayed and progressive establishment pattern during the early neonatal period. However, because of the small sample size, short sampling period, lack of formal source-tracking analysis, and absence of low-biomass negative controls, these findings should be interpreted cautiously as preliminary associations rather than definitive evidence of maternal microbial association. Further longitudinal and strain-resolved studies are needed to determine how maternal microbial exposures affect long-term gut microbial maturation, lamb health, and production performance. 16S rRNA gene sequencing provides relative abundance data and cannot determine absolute bacterial or archaeal loads. Future studies should include qPCR or other quantitative approaches to measure total bacterial and archaeal abundance.

## Figures and Tables

**Table 1:** Dietary ingredients and chemical composition of basal diet (DM basis, %).

Diet Composition		Chemical Composition	
Ingredients	Content %	Items	Nutritional level %
Corn	39.0	Crude protein	12.48
Soybean meal	14.0	Neutral detergent fibre	49.27
Peanut vine	40.0	Crude fat	13.01
wheat bran	30.0		
4% Premix	4.0		

## Data Availability

The original data generated for this study are included in the article. Further inquiries can be directed to the corresponding author.
